# Exploiting vulnerabilities of SWI/SNF chromatin remodelling complexes for cancer therapy

**DOI:** 10.1038/s41388-021-01781-x

**Published:** 2021-05-03

**Authors:** Marek Wanior, Andreas Krämer, Stefan Knapp, Andreas C. Joerger

**Affiliations:** 1 Institute of Pharmaceutical Chemistry, Goethe University Frankfurt, Frankfurt am Main, Germany; 2 Structural Genomics Consortium (SGC), Buchmann Institute for Molecular Life Sciences (BMLS), Frankfurt am Main, Germany; 3 Frankfurt Cancer Institute (FCI), Frankfurt am Main, Germany; 4 German Translational Cancer Network (DKTK) site Frankfurt/Mainz, Frankfurt am Main, Germany

**Keywords:** Targeted therapies, Target identification, Target validation, Chromatin remodelling, Epigenetics

## Abstract

Multi-subunit ATPase-dependent chromatin remodelling complexes SWI/SNF (switch/sucrose non-fermentable) are fundamental epigenetic regulators of gene transcription. Functional genomic studies revealed a remarkable mutation prevalence of SWI/SNF-encoding genes in 20–25% of all human cancers, frequently driving oncogenic programmes. Some SWI/SNF-mutant cancers are hypersensitive to perturbations in other SWI/SNF subunits, regulatory proteins and distinct biological pathways, often resulting in sustained anticancer effects and synthetic lethal interactions. Exploiting these vulnerabilities is a promising therapeutic strategy. Here, we review the importance of SWI/SNF chromatin remodellers in gene regulation as well as mechanisms leading to assembly defects and their role in cancer development. We will focus in particular on emerging strategies for the targeted therapy of SWI/SNF-deficient cancers using chemical probes, including proteolysis targeting chimeras, to induce synthetic lethality.

## Introduction: SWI/SNF chromatin remodelling complexes as potential targets for cancer therapy

For many decades, oncologists have mostly used non- or poorly specific treatment options such as chemotherapy, causing considerable harm to non-cancerous tissues. Recently, new approaches, including immunotherapy and targeted therapy, have increased the number of therapy options for the treatment of some cancers, resulting in a considerable improvement in treatment efficacy and a reduction of side effects. The discovery of new cancer-specific genetic or epigenetic liabilities will help to develop next-generation anti-cancer drugs and personalised medicines.

Epigenetic mechanisms, including chromatin remodelling, control access to a specific genetic locus on chromatin. Epigenetic alterations are involved in the development of many diseases, in particular in tumourigenesis, where dysregulated epigenetic modulators often constitute strong oncogenic drivers [1]. The role of chromatin remodellers in human carcinogenesis has recently been demonstrated in a number of genome-wide and exome-wide sequencing studies, revealing that genes encoding components of the SWI/SNF complex are mutated in 20–25% of human cancers [2]. This high mutation rate indicates tumour suppressive roles, which leads to the question of how perturbations of SWI/SNF chromatin remodellers contribute to tumourigenesis, how they promote tumour growth and, more importantly, whether SWI/SNF complexes can be targeted therapeutically. Targeting chromatin remodelling complexes appears challenging at first glance because many SWI/SNF-associated cancers exhibit loss-of-function mutations due to missense or nonsense mutations, or the complete loss of individual SWI/SNF-subunits [3, 4]. But, functional genomic studies have identified vulnerabilities with other genes causing sustained synthetic lethal interactions [5–7]. Synthetic lethality occurs when the combination of deficiencies in the expression or function of two genes leads to cell death, whereas a deficiency in only one of them results in a viable phenotype [8]. Cancers with SMARCA4 deficiency, a genetic alteration frequently observed in malignant solid tumours or lung adenocarcinomas, for example, show a dependency on the paralogue SMARCA2 [6]. They are also sensitive to perturbations in the SWI/SNF interactome, including protein kinases such as Aurora A [9] or cyclin-dependent kinase 4/6 (CDK4/6) [10]. In this review, we discuss the recent progress in targeting SWI/SNF-mutant cancers with highly specific inhibitors, also called “chemical probes”, as well as FDA-approved drugs that may pave the way for new therapeutic concepts to treat cancer patients.

## Structural assembly and function of SWI/SNF complexes

The human genome consists of three billion base pairs, which corresponds to an approximately two-metre-long DNA molecule that has to be condensed into a tight chromatin structure in order to fit into the nucleus of a cell with a diameter of only 5 µm [11]. The basic units of chromatin are nucleosomes, consisting of 147 base pairs of DNA wrapped around an octamer of four core histones (H2A, H2B, H3 and H4), resulting in a compact chromatin structure. Dense chromatin DNA, termed heterochromatin, is inaccessible to protein binding, with the result that gene expression is largely inactivated. Whenever the chromatin structure is altered to an open euchromatin state through epigenetic mechanisms, such as DNA demethylation or histone acetylation, and the action of chromatin remodellers, transcription factors and associated proteins can access their target genes and initiate gene expression, thereby switching on signalling cascades and biological pathways [11].

**Fig. 1:**
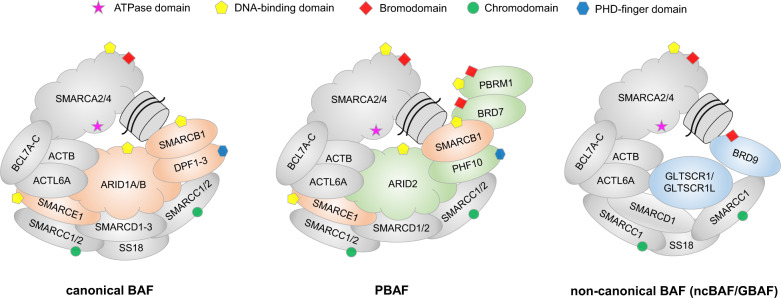
Subunit composition and assembly of mammalian SWI/SNF chromatin remodelling complexes BAF, PBAF and ncBAF. Illustration based on the cryo EM structures of nucleosome-bound BAF and the latest data on SWI/SNF complex assembly [16–18]. Nucleosomes are bound via DNA- and histone-binding domains. Shared SWI/SNF subunits are coloured in grey, BAF subunits not found in ncBAF in orange, PBAF-specific subunits in green and ncBAF-specific subunits in blue.

Four evolutionarily conserved classes of ATP-dependent chromatin remodelling complexes have been discovered in mammalians: ISWI, CHD, INO80 and SWI/SNF [12]. These multiprotein complexes (up to 2 MDa in size) all share a central Snf2-like ATPase domain, but they differ in the composition of interacting subunits and recruited proteins, which determines their biological roles (Fig. 1, Table 1) [12, 13]. SWI/SNF complexes, for example, consist of a combinatorial product of at least 29 proteins. The discovery of SWI/SNF chromatin remodellers and their evolutionary history have been described in detail elsewhere [13]. SWI/SNF complexes are essential for cell proliferation and differentiation, but the exact mechanisms of how these remodellers carry out their role are currently poorly understood. They contain several nucleosome-binding domains, including DNA-binding domains, such as AT-rich interactive domains (ARIDs), zinc-finger domains or high-mobility group box domains (HMGs), and histone-binding domains, such as bromodomains (BRDs), plant homeodomains (PHDs) and chromodomains, which are found in most SWI/SNF subunits (Fig. 1, Table 1) [12, 14].

**Table 1:** Binding interactions/functions of SWI/SNF subunits and their role in cancer.

Complex	SWI/SNF subunits (*genes*)	Domains^a^	Chromatin interaction	SWI/SNF-associated cancer
BAF/PBAF/ncBAF	BRG1 (*SMARCA4*)	ATPase, helicase, bromodomain BRK, HSA, QLQ, SnAC [130]	DNA binding, histone binding	Breast cancer, cutaneous cancer, B-cell lymphoma, bladder carcinoma, non-small cell lung cancer, melanoma, glioma carcinoma,
	BRM (*SMARCA2*)	ATPase, helicase, bromodomain, BRK, HSA, QLQ, SnAC	DNA binding, histone binding	Ovarian small cell carcinoma, sarcoma, lung adenocarcinoma, hepatocellular carcinoma
	BAF53A (*ACTL6A*)	Actin-like	Not reported	Squamous cell carcinoma, rhabdomyosarcoma
	SS18* (*SS18*)	No domain annotation	Not reported	Synovial sarcoma, Ewing sarcoma
	β-actin (*ACTB*)	Armadillo repeats	Not reported	Abnormal expression and polymerisation of ACTB in most cancers leading to changes of the cytoskeleton associated with invasiveness and metastasis [133].
	BAF155 (*SMARCC1*)	Chromodomain, SWIRM, SANT	Histone binding	Rhabdoid cancer, small cell lung caner
	BAF170^§^ (*SMARCC2*)	Chromodomain, SWIRM, SANT	Histone binding	Gastric cancer, colorectal cancer, rhabdoid cancer, pancreatic cancer
	BCL7 (A, B or C) (*BCL7A*, *B* or *C*)	Conserved N-terminal domain	Not reported	Multiple myeloma, non-Hodgkin lymphoma
	BAF60 (A, B^§^ or C^#^) (*SMARCD1*,*2* or *3*)	SWIB	Not reported	Lung cancer, gastric cancer
	BAF57^§^ (*SMARCE1*)	HMG box	DNA binding	Breast cancer, endometrial carcinoma, ovarian carcinoma, meningioma
	BAF47^§^ (*SMARCB1*)	Winged helix DNA-binding domain [34, 35]	DNA binding, nucleosome acidic patch binding	Malignant rhabdoid tumour, prostate cancer, renal medullary carcinoma, epithelioid sarcoma, familial schwannomatosis
BAF	BAF250 (A or B) (*ARID1A* or *B*)	ARID (AT-rich interactive domain)	DNA binding	Ovarian clear cell carcinoma, uterine endometrial cancer, neuroblastoma, colorectal cancer
	BAF45 (B, C or D) (*DPF1,2 or 3*)	Zinc finger/PHD finger	Histone binding	Colorectal cancer
PBAF	BAF200 (*ARID2*)	ARID, RFX winged helix DBD, zinc finger	DNA binding	Melanoma, non-small cell lung cancer, hepatocellular carcinoma, colorectal carcinoma
	BRD7 (*BRD7*)	Bromodomain, DUF3512	Histone binding	Hepatocellular carcinoma, nasopharyngeal carcinoma, lung cancer, ovarian carcinoma
	BAF180 (*PBRM1*)	Bromodomain, BAH domain, HMG box	DNA binding, histone binding	Clear cell renal cell carcinoma
	BAF45A (*PHF10*)	PHD finger	Histone binding	Gastric cancer
ncBAF	BRD9 (*BRD9*)	Bromodomain, DUF3512	Histone binding	Nut midline carcinoma, rhabdoid cancer
	GLTSCR1 (*BICRA*)	No annotation	Not reported, BRD4 interactor	Glioma tumour, prostate cancer
	GLTSCR1L (*BICRAL*)	No annotation	Not reported, BRD4 interactor	Prostate cancer

^a^Selected domains annotated in UniProt or the indicated reference. *Found only in BAF and ncBAF. ^§^Found only in BAF and PBAF. ^#^Found only in BAF.

**Fig. 2:**
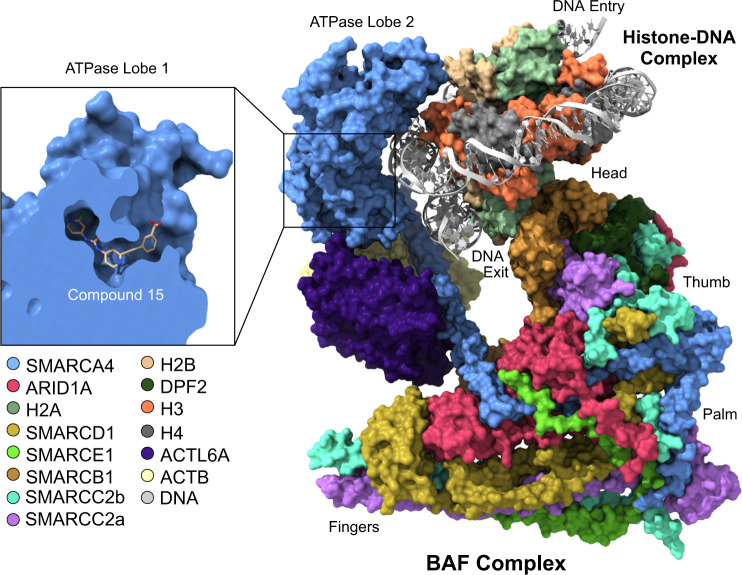
Cryo-EM structure of human BAF in complex with nucleosome core particle. Individual subunits are shown in different colours (PDB entry 6LTJ) [17]. ARID1A serves as the core of the base module stabilising the entire complex, while SMARCA4 and SMARCB1 bind the nucleosome. The insert shows the ATPase domain of SMARCA4 in complex with an allosteric ATPase inhibitor (PDB entry 6EG3) [74].

Three mammalian SWI/SNF complexes have been identified: BAF (BRG1/BRM-associated factor), PBAF (polybromo-associated BAF) and, very recently, non-canonical BAF (ncBAF or GBAF) [15, 16]. They all contain a mutually exclusive catalytic ATPase subunit, SMARCA2 or SMARCA4, and share a number of associated proteins (Fig. 1, Table 1). BAF and PBAF, for example, both contain an ARID and a SMARCB1 subunit. The recently published cryo electron microscopy (EM) structures of recombinant and endogenous human BAF complexes have provided exciting new insights into the overall assembly of the complex and the mechanisms of how nucleosomes interact with BAF (Fig. 2) [17, 18]. These structures revealed a modular organisation of the complex that is consistent with earlier data on the complex assembly pathway [16], and also provided the first mechanistic insights into ATP-driven rearrangements causing histone eviction. ARID1A forms the structural core of the base module, with SMARCC1/2 serving as scaffolding proteins and SMARCE1/D1 supporting complex formation. The nucleosome is sandwiched between SMARCB1 and the ATPase module SMARCA4, which both interact with nucleosome H2A/H2B acidic patch regions. ATP-driven engagement of SMARCA4 with the nucleosome eventually leads to nucleosome eviction, creating an elongated stretch of nucleosome-free DNA that is accessible for transcription factors.

PBAF, the second main SWI/SNF chromatin remodeller in humans, shares high homology with BAF, but there are also significant differences in its subunit composition [16]. ARID1A, the structural core of the base module of the BAF complex is replaced in PBAF by the paralogue ARID2. In addition, PBAF contains the bromodomain-containing protein BRD7. A particularly interesting feature that is unique to PBAF and hence gives it its name: the subunit PBRM1 (polybromo-1), which contains six tandem-acting bromodomains (PB1(1-6)). In the smaller ncBAF, the central ARID1A subunit of BAF is replaced by the glioma tumour suppressor candidate region gene 1 (GLTSCR1) subunit [15]. ncBAF further lacks other base module BAF subunits, including SMARCC2, SMARCE1 and the nucleosome-recognition unit SMARCB1, but features the bromodomain-containing BRD9 subunit, which is not present in the other two complexes, according to the most recent systematic study of SWI/SNF complex assembly [16].

Due to their combinatorial and hence structural diversity, the three human SWI/SNF chromatin remodellers interact specifically with various enhancers and promoters in a cell-type-specific manner. BAF complex subunits also undergo distinct switches during development, resulting in diverse transcriptional signatures [19]. BAF is generally associated with binding to enhancers, whereas PBAF or ncBAF are frequently enriched at promoter regions [20, 21]. SMARCA4, for example, is known to co-localise with H3K27ac at enhancers regulating lineage-specific developmental programmes [22]. Two recent publications have provided intriguing insights into how expression levels of a key subunit, in this particular case SMARCB1, which is unique to BAF and PBAF, can shift the balance between canonical and non-canonical SWI/SNF complexes and hence alter transcriptional signatures. SMARCB1 loss results in a widespread impairment of typical enhancer activity but not super-enhancer activity [23]. Conversely, SMARCB1 negatively regulates super-enhancers in any cell type, suggesting that high levels of SMARCB1 prevent the formation of ncBAF [24].

## Role of SWI/SNF complexes in cancer development

SWI/SNF complexes play a fundamental role in maintaining and regulating the access of transcription factors, and, therefore, they also exert considerable tumour-suppressive activities. Consequently, SWI/SNF perturbations can trigger reprogramming of cellular processes and drive oncogenic programmes. Loss-of-function mutations in genes encoding SWI/SNF subunits are found in >20% of human cancers, with point mutations occurring about twice as often as deletions [25]. The recently published cryo EM structures of the human BAF complex revealed that a large fraction of oncogenic mutations maps to intra-complex subunit–subunit interfaces, exposed surfaces that may interact with regulatory proteins or interaction sites with the nucleosome, thereby altering the chromatin remodelling activity of the complex [18]. SWI/SNF genes are also amplified in many cancers. The prevalence of amplifications strongly depends on the type of cancer and occurs most commonly in lung squamous cell carcinoma, ovarian cancer and sarcoma [25]. Especially BRD9 and ACTL6A show a high amplification frequency across multiple cancers, highlighting their oncogenic potential [25].

SWI/SNF chromatin remodellers were first linked to cancer development more than two decades ago when it was discovered that biallelic loss-of-function mutations of the SMARCB1 gene drive tumourigenesis in malignant rhabdoid tumours (MRTs) [26]. The main reason for developing MRT is that SMARCB1 loss prevents interaction of BAF with typical enhancers, whereas binding to super-enhancers is barely affected [23]. These observations led to the assumption that the remaining SWI/SNF subunits induce gene expression mainly at super-enhancers but not at typical enhancer sites, thereby maintaining aberrant cell identity and enabling MRT survival [23]. Newer studies have now provided a more detailed picture, showing that gene expression in MRT is driven by the BRD9-containing ncBAF complex, which does not require a SMARCB1 subunit [18, 21, 27, 28]. In addition, SMARCB1 rescue experiments in MRTs restored enhancer-based activation and demonstrated that wild-type BAF is able to antagonise polycomb repressive complex mediated gene repression [20]. These findings are consistent with an earlier study showing that SMARCB1 loss leads to an increased expression of the polycomb subunit EZH2 (enhancer of zeste 2) and to elevated levels of H3K27me3 gene silencing marks during MRT transformation [29].

Loss of SMARCB1 activity is also a major driver for developing aggressive synovial sarcoma (SS). In the development of this tumour type, a chromosomal translocation involving BAF subunit SS18 leads to the formation of an SS18-SSX fusion protein [30] that displaces SMARCB1 in the BAF complex. As a result, SS18-SSX-containing BAF is retargeted from specific enhancer regions, inducing the activation of a unique transcriptional signature in SS [31]. SMARCB1 loss has been linked to the activation of several oncogenic signalling pathways (e.g., hedgehog or Wnt) and the development of malignant tumours such as renal medullary carcinoma [32, 33]. Mutations in the N-terminal winged-helix DNA-binding domain of SMARCB1 have been associated with schwannomatosis, a syndrome predisposing to mostly benign tumours of the central nervous system [34, 35].

SMARCA4 is the most frequently mutated Snf2-like ATPase in humans [36], and it has been found to be inactivated or disrupted in many cancers, including breast cancer [37], lung cancer [38] and colorectal cancer [39]. Although the ARID1 subunit of BAF and the PBRM1 subunit of PBAF exhibit the highest mutation prevalence, SMARCA4-mutant cancers are typically more aggressive and are associated with a poorer prognosis [36]. The ATPase subdomain of SMARCA4 is a mutational hotspot. Mutations of this subdomain not only result in impaired ATPase activity and nucleosome remodelling [36] but also impair BAF competition with polycomb repressive complexes [40]. In contrast, the SMARCA4 paralogue SMARCA2 is less frequently mutated in cancer. It is, however, frequently epigenetically silenced, which has been demonstrated, for example, by restoring SMARCA2 function in SMARCA2-deficient cancers using histone deacetylase (HDAC) inhibitors [41].

The BAF subunit ARID1A binds DNA non-specifically via its AT-rich interactive DNA-binding domain and has the highest mutation frequency among BAF subunits in cancer [42]. ARID1A acts as a tumour suppressor, and loss of function has been associated with tumour development in ovarian carcinoma [43], pancreatic cancer [44] and cervical cancer [45]. Interestingly, the cancer mutations are spread across almost all regions of the ARID1A gene and do not cluster in the DNA-binding domain as seen in transcription factors such as p53 [42, 46]. One reason for this is that ARID1A is involved in many subunit–subunit contacts stabilising the base module of the complex that are susceptible to inactivating mutations. Loss of ARID1A tumour suppressive functions triggers cancer development through perturbations of DNA-damage response and cell-cycle pathways [42]. Furthermore, several studies have also demonstrated that an ARID1A loss is associated with activation of phosphatidylinositol-4,5-bisphosphate 3-kinase catalytic subunit alpha (PIK3CA) and a concurrent loss of PTEN expression, which both activate the PI3K/AKT/mTOR cell-cycle pathway [47–50].

ARID1B-related cancers are less common. Mutations of this gene, however, have been associated with developmental disabilities such as the Coffin-Siris syndrome [51, 52]. There is compelling evidence, though, that its paralogue in PBAF, ARID2, acts as a tumour suppressor. ARID2 is frequently mutated in non-small cell lung carcinoma [53] and hepatocellular carcinoma [54]. Mechanistic studies on the role of ARID2 in hepatocellular carcinoma and lung cancer have shown that an ARID2 loss drives transcriptional reprogramming and impairs the DNA-damage response pathways [55, 56].

PBRM1 is mutated in ~40% of clear cell renal carcinomas, which makes it the second most frequently mutated tumour suppressor gene in kidney cancer after the von Hippel-Lindau protein (VHL) [57]. Interestingly, only a concurrent loss of PBRM1 and VHL induced renal carcinogenesis in a preclinical mouse model [58]. Mechanistic studies revealed that PBRM1 is enriched at promoter sites flanked by H3K14ac marks and that PBAF is recruited to the chromatin by the tandem-acting bromodomains of PBRM1 [59, 60]. This chromatin engagement seems to be very tight, as only a triple-mutant variant inactivating the second, fourth and fifth PB1 bromodomain caused re-localisation of PBRM1 to the cytoplasm [61–63]. However, overexpression of PBRM1 failed to suppress tumour growth in a mouse xenograft model.

Taken together, these studies highlight that components of SWI/SNF complexes are mutated in a multitude of cancers, either promoting oncogenic mechanisms or inactivating tumour suppressor functions. SWI/SNF components should therefore be high-priority targets for cancer therapy. Unfortunately, loss-of-function mutations are difficult to target, which is why they are often neglected as therapeutic targets in drug discovery.

## Exploiting SWI/SNF vulnerabilities for targeted cancer therapy

Within the Achilles project (http://www.broadinstitute.org/achilles), systematic genetic knockout experiments of individual SWI/SNF subunits or SWI/SNF-regulating proteins identified a number of promising genes for targeted therapy of SWI/SNF-mutant cancers (Table 2) [5–7, 64–67]. One therapeutic strategy targets the epigenetic antagonism between SWI/SNF complexes and the polycomb repressive complexes, which has been excellently reviewed in detail elsewhere [68]. Another approach exploits synthetic lethal interactions, where a concurrent loss of two genes leads to cell death, while a single perturbation of one of these genes is viable.

**Table 2:** Synthetic lethal interactions and chemical tools for the targeted therapy of SWI/SNF-mutant cancers.

SWI/SNF-deficiency	Synthetic lethal interaction/ epigenetic antagonism	Inhibitors	Ref.
SMARCA4	SMARCA2	PFI-3^#^, ACBI1^#^, ATPase-14^#^	[6, 65, 66]
	ACTB	n/a	[5]
	ARID2	n/a	[5]
	CDK4/6	Palbociclib^§^, Abemaciclib^§^, Ribociclib^§^	[10, 111]
	Aurora A	Tozasertib* (VX-680)	[9]
	OxPhos	IACS-010759*	[134]
	BET	( + )-JQ1^#^, OTX-015*	[135]
	EZH2	Tazemetostat^§^, GSK126*, CPI-169^#^	[136–138]
ARID1A	ARID1B	n/a	[7, 64]
	EZH2	GSK126*	[136, 139]
	PARP	Talazoparib^§^, Olaparib^§^, Rucaparib^§^, Veliparib^§^	[119, 120]
	PI3K/AKT	MK-2206*, Perifosine*, Buparlisib*	[49]
	ATR	VE-821^#^, VX-970 (M6620)*	[98]
	Abl, Src, c-KIT	Dasatinib^§^	[110]
	GCLC	Buthionine sulfoximine*	[128]
	Aurora A	TCS-7010^#^	[108]
	HDAC6	Ricolinostat (ACY-1215)*	[114]
SMARCC1	SMARCC2	n/a	[5]
SMARCA2	SMARCA4	dBRD9^#^	[140]
PBRM1	EZH2	GSK126*	[136, 141]
ARID2	PARP	Veliparib^§^	[56]
SMARCB1	EZH2	Tazemetostat^§^	[29]
	HDAC	Panobinostat^§^	[112]
	BRD9	BI-7273^#^/9564^#^, dBRD9^#^, VZ185^#^	[21, 27]
	MDM2/4	Idasanutlin*	[121]
	UBE2C	Ixazomib^§^, bortezomib^§^	[123]
SS18-SSX	BRD9	dBRD9^#^	[27, 28]
	KDM2B	n/a	[124]
	ATR	VX-970*, AZD6738*	[142]

^#^Chemical probe. *Clinical probe. ^§^FDA-approved drug.

A number of intra- and inter-complex SWI/SNF vulnerabilities have been identified that are associated with a synthetic lethal phenotype in SWI/SNF-deficient cancers (Table 2). In some cases, synthetic lethality is caused by the concomitant knockout of two mutually exclusive paralogues [69]: Tumours with a SMARCA4 deficiency are sensitive to SMARCA2 depletion [65], and ARID1B is required for survival of ARID1A-mutant cancers [7]. In addition, BRD9 is a specific vulnerability in SMARCB1-deficient malignant rhabdoid tumours and in synovial sarcoma (SS18-SSX) cells [21, 27, 28]. This particular synthetic lethality can be rationalised based on the composition and structure of the three types of SWI/SNF complexes outlined above (Fig. 1). The assembly and structural integrity of canonical BAF and PBAF complexes depend on SMARCB1, whereas assembly and function of ncBAF do not require SMARCB1. Correct assembly and integrity of ncBAF, which drives oncogenic signalling in MRT, however, depends on the scaffolding function of BRD9 [16]. So, the additional knockdown of BRD9 in SMARCB1-deficient cells also abrogates the function of the remaining non-canonical SWI/SNF chromatin remodelling complexes and is therefore lethal. Additional vulnerabilities have been recently identified in a large genetic screen, revealing intra-complex synthetic lethal interactions between SMARCC1/SMARCC2, SMARCA4/ACTB and SMARCA4/ARID2 [5].

Vulnerabilities of SWI/SNF-mutant cancers have also been found in the interactome of SWI/SNF chromatin remodellers, including protein kinases involved in cell-cycle control or DNA-damage repair (Table 2). Although not every protein kinase has been targeted to date, there is a large diversity of inhibitors available for every family of the kinome that could provide excellent starting points for targeted therapy. Targeting specific SWI/SNF vulnerabilities with small molecules is therefore a promising strategy for this highly complex system. However, this approach requires the identification and validation of druggable targets and the development of high-quality chemical probes to elucidate the biological role of a target of interest [70]. Such probes must fulfil a number of specific criteria, including chemical stability, selectivity and potency, and their mode of action must be sufficiently well characterised [71, 72].

## Direct targeting of SWI/SNF components for inducing synthetic lethality

The development of effective chemical probes for the direct targeting of components of SWI/SNF complexes to exploit synthetic lethal interactions relies on the presence of unique and well-defined druggable pockets. Targeted SWI/SNF drug discovery has therefore mainly focused on the ATPase and bromodomains. SMARCA2/4 contains a bromodomain and an ATPase domain, BRD7 and BRD9 contain a bromodomain, and PBRM1 has six tandem-acting PB1 bromodomains. Targeting these domains may be desirable in the context of a synthetic lethal approach to inactivate specific SWI/SNF subunits but is of course also a promising strategy to neutralise an over-expression and/or aberrant oncogenic function of SWI/SNF subunits. A prime example is BRD9, which is often amplified in cancers [25] but is also a specific vulnerability in SMARCB1-deficient cancers [21, 27, 28]. In the following, we will first describe the structural features and druggability of bromodomain and ATPase modules and then discuss the current state of chemical probe development and its implications.

### Structure and druggability of SWI/SNF ATPase and bromodomains

The ATPase domain of human SWI/SNF complexes is an Snf2-like ATPase that uses the energy from ATP hydrolysis to remodel the chromatin structure. Until very recently, most structural information was based on Snf2 homologues from non-human species, though [73]. The cryo-EM structures of human BAF have now provided exciting mechanistic insights into BAF assembly and potential mechanisms for nucleosome/DNA unwinding [17, 18]. Due to the high flexibility of the SMARCA4 subunit within the BAF complex, the resolution of this particular region of the complex was relatively poor, which prevented a detailed view of the structure of the ATPase domain in the context of the overall complex [17]. It was, however, possible to determine the crystal structure of the N-RecA lobe of the human SMARCA2 ATPase domain, which revealed a druggable allosteric pocket in the proximity of the ATP-binding site (Fig. 2) [74].

Bromodomains are highly conserved protein–protein interaction modules that recognise acetylated lysines on histone tails. The human genome encodes 61 BRDs that are part of 46 larger multi-domain proteins. They can be subdivided into eight families, which are mainly associated with DNA accessibility and the control of gene regulation [75]. Bromodomain-mediated transcriptional activation is often enhanced by the concurrent interaction with additional chromatin-modifying enzymes to ensure accurate chromatin engagement and gene expression. All BRDs comprise ~120 amino acids and fold into a characteristic structure that contains a bundle of four α-helices (αA, αB, αC, αZ) with a hydrophobic cavity formed by two loops (ZA, BC). In canonical BRDs, acetylated lysines (Kac) are anchored via a highly conserved asparagine and a network of water molecules [75]. The biological substrate specificity is mainly determined by the electrostatic potential of the BRD surface. The bromodomains BRD7/9, SMARCA2/4 and PB1(2-5) read histone acetylation marks such as H3K14ac [63], H3K27ac [22] or H3K9ac [76], thereby recruiting SWI/SNF chromatin remodelling complexes to chromatin.

**Fig. 3:**
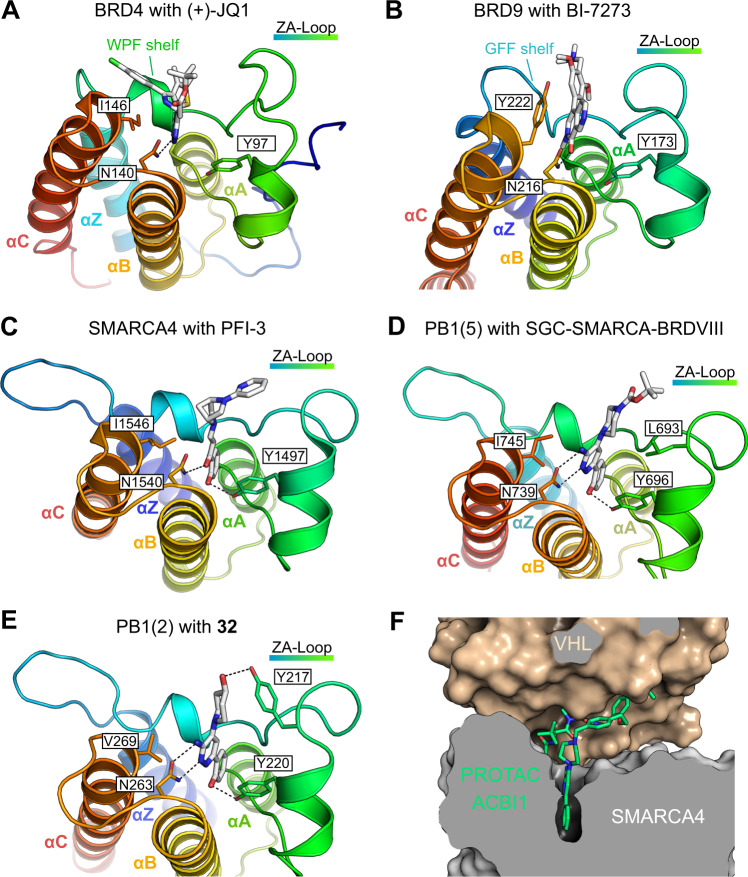
Druggable binding pockets of SWI/SNF chromatin remodelling complex bromodomains with selected chemical probes. **A**, **B** Comparison of BRD4 in complex with (+)-JQ1 (PDB entry 3MXF) [82] and BRD9 in complex with BI-7273 (PDB entry 5EU1) [80] reveals a different shape of the binding pocket. The residue numbering in the BRD9 structure is based on the canonical isoform in UniProt entry Q9H8M2, which differs from the numbering in the original PDB entry that is based on an isoform lacking the first 116 residues. **C**–**E** Crystal structures of BRD subfamily VIII members with bound inhibitors (PDB entries 5DKD, 6ZS4, 6ZNV) reveal a similar overall inhibitor binding mode [90, 93]. **F** Crystal structure of the ternary complex of SMARCA4 and the E3 ubiquitin ligase VHL bound to PROTAC ACBI1, showing that the complex is stabilised by additional protein–protein interactions (PDB entry 6HR2) [104].

The members of BRD subfamily IV, BRD7 and BRD9, share a high sequence identity and form a highly conserved overall structure, especially in the Kac binding site, but some notable minor differences are found in the vicinity of the binding pocket [77]. As a consequence, the biological roles of BRD7 and BRD9 are remarkably different, which may explain their mutual exclusivity in either PBAF or ncBAF, respectively [78, 79]. This phenomenon of different substrate specificities of BRD family members is exemplified by comparing BRD9 with BRD4, a member of the bromodomain and extra-terminal domain (BET) family. BRD9 has a different shape of the binding pocket because the gatekeeper residue Tyr222 on helix αC blocks the entrance to a subpocket, which is accessible in BRD4 (WPF shelf region) due to a smaller hydrophobic residue at this position (Ile146) (Fig. 3a, b) [80].

In members of BRD subfamily VIII, SMARCA2/4 and PB1(1-6), the binding pocket is smaller and lacks well-defined hydrophobic grooves. They also have a shorter ZA loop than BRD4, somewhat reducing potential ligand interactions with this region (Fig. 3A, C–E) [81]. SMARCA2/4 and PB1(2-5) are canonical BRDs, whereas the first and sixth BRD of PBRM1 have either an occluded binding site or lack the conserved asparagine. The PB1(2-4) BRDs additionally have a specific tyrosine in the ZA loop that acts as a lid at the entrance of the binding pocket [81].

### Chemical probes for targeting individual SWI/SNF domains

**Fig. 4:**
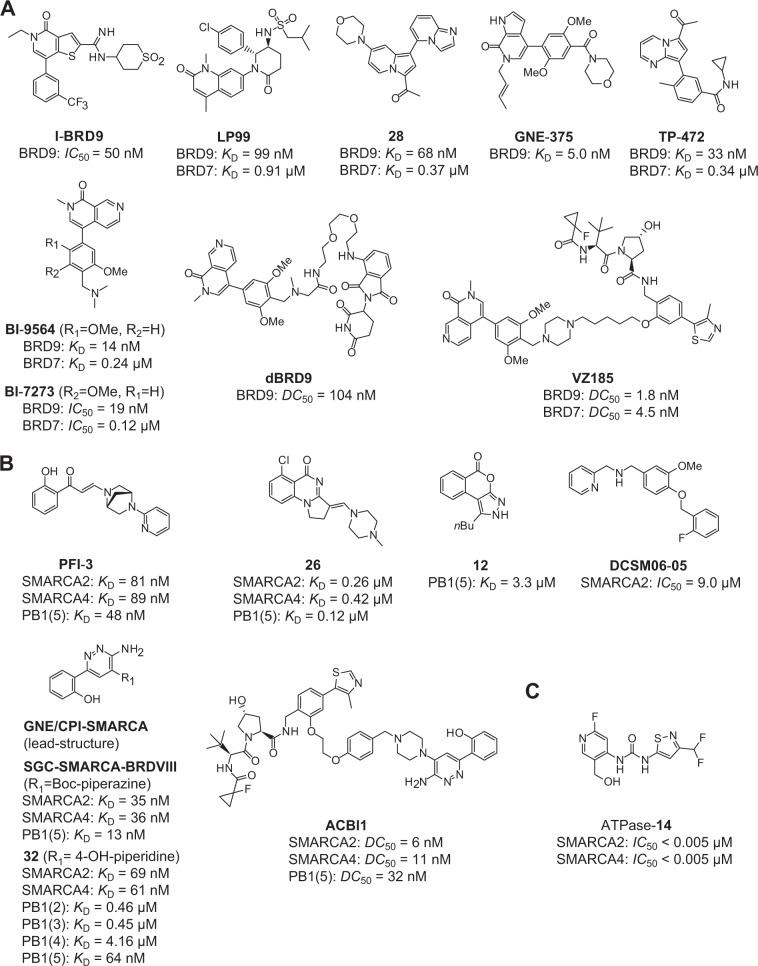
Selection of small molecules targeting the SWI/SNF bromodomains and the ATPase domain. **A** Inhibitors and PROTACs targeting bromodomains BRD7/9 [80, 86–89, 102, 103]. **B** Inhibitors and PROTACs targeting the bromodomains of SMARCA2/4 and PBRM1 [90–95, 104]. **C** Allosteric inhibitor of the ATPase domain of SMARCA2/4 [74].

Early bromodomain drug discovery focused on the development of chemical probes targeting the BET bromodomains (bromodomain subfamily II) [82–84]. More recently, an increasing number of new bromodomain inhibitors targeting other bromodomain families have been reported, especially chemical probes targeting the bromodomains of SWI/SNF complexes [85]. For example, many BRD7/9 inhibitors are now available to study the biological roles of these essential epigenetic modulators (Fig. 4A). However, due to the high sequence conservation of the binding pockets of BRD7/9, most of the available chemical probes exhibit dual BRD7/9 activity [77, 80, 86–88]. The first published selective dual BRD7/9 inhibitor, LP99, was developed from a quinolone-fused lactam in an SAR study [86]. Shortly after, a selective BRD9 inhibitor, I-BRD9, was reported with a 200-fold selectivity over BRD7 [89]. The selectivity was attributed to small differences in the GFF shelf region (GFF vs. AFF in BRD7) and the ZA channel of the two bromodomains.

The first published SMARCA2/4 and PB1(5) bromodomain inhibitor, PFI-3, was developed in an SAR study using an unusual salicylic acid fragment as an acetyl-lysine mimetic moiety (Figs. 3c and 4b) [90]. Structural studies showed that PFI-3 penetrated deeper into the binding pocket compared with the canonical binding mode of earlier BRD inhibitors by displacing conserved structural water molecules, resulting in an excellent selectivity profile within all BRD families. Due to the lack of chemical stability of PFI-3 in cellular systems over longer time periods, several other SMARCA2/4 BRD inhibitors were developed [91, 92]. However, only one of them, SGC-SMARCA-BRDVIII (compound **22**) [93], which has been recently developed based on a patent [94], met chemical probe criteria in terms of potency (Figs. 3d and 4b). The PB1 BRDs have not been considered as drug targets so far. Due to the high sequence conservation in the acetyl-lysine binding site of BRD subfamily VIII, most of the PB1-BRD inhibitors show selectivity for the fifth PB1 bromodomain and for SMARCA2/4 BRDs; the recently published compound **32**, however, also showed activity on the second and third bromodomain of PB1 (Figs. 3e and 4b) [90, 91, 93, 94]. Only one selective ligand with low affinity for the fifth PB1-BRD has been developed so far [95].

Besides targeting the BRDs, an allosteric inhibitor of the ATPase domain of SMARCA2/4 was developed (Fig. 4C) [74]. High throughput screening methods and hit validation techniques identified a dipyridyl urea derivative as initial lead compound, which was subsequently developed into several inhibitors (Fig. 4C). This inhibitor class binds to an allosteric pocket in the vicinity of the ATP binding site, with the two NH groups of the urea moiety interacting with the carboxylate group of the catalytic residue Glu852 [74].

Other than that, no additional direct-acting inhibitors for other subunits of SWI/SNF complexes have been developed so far because of a lack of known binding pockets. However, a non-toxic 12-membered macrolactam, BRD-K98645985, was recently identified in a high-throughput screen for inhibitors of BAF-mediated transcription in cells, which seems to preferentially bind to ARID1A-containing BAF complexes and relieves transcriptional repression of HIV-I [96]. This compound was further found to synergize with ATR (ataxia-telangiectasia mutated (ATM) and Rad3-related protein kinase) inhibitor VE-821 and induce a synthetic lethal interaction in cancer cells [97], which had been described before in RNA interference screens [98]. The binding site or the specific target protein of BRD-K98645985 remains elusive.

### Phenotypic response of direct SWI/SNF domain inhibition and the emergence of PROTACs as a game-changing technology

The excitement that specific bromodomain inhibitors are available to potentially exploit synthetic lethal interactions in SWI/SNF mutant cancers was initially somewhat dampened by their failure to induce synthetic lethality. The first example to illustrate this issue are the SMARCB1-deficient cancers that are sensitive to depletion of the ncBAF subunit BRD9. Initial biological assays with inhibitor I-BRD9 indicated that BRD9 bromodomain function is not required for cell proliferation but regulates gene expression associated with oncogenic and immunoresponsive pathways [89]. The chemical probes I-BRD9 and BI-7273 both showed some weak cytotoxic effect in SMARCB1-deficient MRT and synovial sarcoma but, importantly, only at a relatively high compound concentration of about 10 µM or higher, hinting at off-target activity of these inhibitors at high concentration [99]. Overall, targeting BRD7/9 with conventional BRD inhibitors failed to fully recapitulate the phenotypic response of genetic BRD9 knockout studies in SMARCB1-deficient cancers. Experiments with diverse BRD9 truncation mutants revealed that its bromodomain is dispensable for the integrity of ncBAF, and that the yet undruggable, poorly characterised DUF3512 domain has an essential scaffolding function and constitutes the actual vulnerability in MRT [21]. The initial disappointment, however, soon disappeared with the emergence of the first bromodomain-specific degraders [100]. Proteolysis-targeting chimeras, PROTACs, may represent a game-changing technology, as this new class of small-molecule modulators initiate the degradation of a protein of interest rather than merely inhibiting the function of a specific domain [101]. By linking a conventional inhibitor to an E3 ligase recruiting moiety (e.g. thalidomide or VHL ligand 1), PROTACs induce ubiquitin transfer onto target proteins, thereby marking them for proteasomal degradation. This technology was applied to target BRD9, and the developed PROTACs dBRD9 [102] and VZ185 [103] (Fig. 4A) were much more effective in treating SMARCB1-mutant cancers than their parent inhibitor BI-7273. dBRD9 is a selective cereblon-based degrader of BRD9, whereas VZ185 targets both BRD7 and BRD9 for proteasomal degradation mediated by the VHL E3-ubiquitin ligase. Both dBRD9 and VZ185 showed increased cytotoxicity in acute myeloid leukaemia and MRT cells that were sensitive to BRD9 depletion. dBRD9 also altered transcriptional programmes essential for SMARCB1 loss-of-function driven tumours, thereby blocking proliferation of synovial sarcoma and MRT cells [27, 28].

The recent progress in the field of targeted degradation also led to the development of ACBI1, a VHL-dependent degrader of SMARCA2/4 and PBRM1 based on the bromodomain inhibitor GNE/CPI-SMARCA (Fig. 4B) [104]. A sensitivity to SMARCA2 loss by RNA interference knockdown was observed in SMARCA4-deficient lung cancers, which triggered a phenotypic lethal response [105]. Inhibition of the SMARCA2 BRD with the chemical probe PFI-3 [90], however, was unable to phenocopy this synthetic lethal interaction [105], but it had pronounced effects on embryonic and trophoblastic stem cell differentiation [106]. The lack of efficacy of SMARCA2/4 BRD inhibitors in SMARCA4-mutated cancers suggested the ATPase domain rather than the BRD as the therapeutically relevant target in oncology, which was confirmed by genetic complementation studies [105]. Consistent with these complementation studies, expression of SMARCA2-associated genes was downregulated and cell growth of SMARCA4-mutant lung cancers was impaired upon inhibition of the ATPase domain with the SMARCA2/4 ATPase inhibitor **14** [74]. The PROTAC ACBI1 has now led to a renaissance of bromodomains as drug targets in SMARCA4-deficient cancers. This compound induced anti-proliferative effects and apoptosis in SMARCA4-mutant cells by triggering proteasomal degradation of the full-length SMARCA2 protein, thereby also knocking out the SMARCA2 ATPase domain [104]. This study further provided insights into the structural basis of protein degradation by elucidating the structural details of the ternary complex between PROTAC-bound SMARCA2 and VHL (Fig. 3F). More recently, an additional PROTAC based on this scaffold targeting SMARCA2/4 was reported [107]. The potential of altering selectivity profiles is another exciting aspect of PROTACs development because efficient PROTAC-mediated degradation depends on the exact geometry and spatial arrangement of the ternary complex with the E3 ligase, which is influenced by regions outside the canonical inhibitor binding site. It may therefore be possible to convert dual or pan-selective inhibitors into selective degraders that exclusively target one subunit type or a particular paralogue, e.g. SMARCA2 or 4, for efficient degradation. At the moment, however, such strategies often rely on serendipity rather than on actual structure-based design.

The above examples highlight the enormous potential of PROTACs for targeting undruggable components of SWI/SNF complexes. Future strategies may aim to develop PROTACs that target non-essential but druggable components of SWI/SNF complexes to eliminate ‘undruggable activities’ in a specific disease context, either to induce synthetic lethality or to counteract the effect of gene amplification.

## Small-molecule modulators for targeting the SWI/SNF interactome

**Fig. 5:**
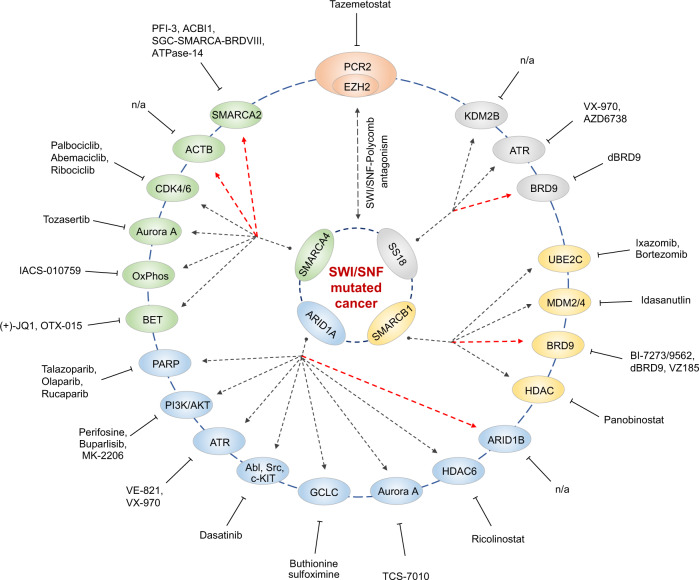
Overview of synthetic lethal interactions in SWI/SNF-mutant cancers with chemical tools for targeted cancer therapy. Known synthetic lethal interactions in SWI/SNF-mutant cancers are shown in matching colours. In addition, the SWI/SNF-polycomb (PCR2/EZH2) epigenetic antagonism pathway is highlighted. Available chemical tools for each target involved in a synthetic lethal interaction in SWI/SNF-mutant cancers are listed. The red arrows highlight synthetic lethal interactions with other SWI/SNF chromatin remodelling complex subunits, whereas the black arrows mark reported vulnerabilities with other genes.

Over the past years, great progress has been made in the identification of synthetic lethal targets that interact with or regulate SWI/SNF chromatin remodelling complexes (Table 2 and Fig. 5). Although not all of these targets have been drugged yet, there is a large collection of inhibitors targeting these proteins described in the literature that may be used to recapitulate the effects of genetic knockdown studies. One exploitable class of proteins are protein kinases, which are involved in many signalling pathways, including cell-cycle control and DNA-damage repair, and for which a myriad of different inhibitors are available. A second targetable protein class are the HDACs, which alter the histone code that is read by chromatin-interacting proteins such as the SWI/SNF bromodomains. A third target group are the poly ADP ribose polymerase (PARP) enzymes, which play a role in DNA-damage repair by modulating chromatin structure through ADP ribosylation. Last but not least, the ubiquitin-ligase/proteasome system as well as the p53/MYC-signalling pathway can be targeted with inhibitors of the p53-MDM2 interaction or the proteasome.

### Protein kinases

Protein kinases are exploitable vulnerabilities in SMARCA4- and ARID1A-mutant cancers. Non-small cell lung cancer (NSCLS) cells carry a SMARCA4 loss-of-function mutation, which makes them highly sensitive to inhibition or deletion of Aurora A, a kinase essential for cell division during mitosis and for the regulation of chromosome segregation. Aurora A inhibition in NSCLS cells with tozasertib, for example, resulted in a synthetic lethal response by reprogramming centrosome pathways during mitotic spindle formation [9]. A recent publication also suggested Aurora A as a potential therapeutic target in ARID1A-deficient colorectal cancer cells [108]. Wild-type ARID1A was shown to downregulate the expression of Aurora A, while ARID1A loss led to increased expression levels of Aurora A and sustained activation levels of cell division cycle 25 C (CDC25C). Inhibition of Aurora A with a pan-selective kinase inhibitor, TCS-7010, in ARID1A-mutant colorectal cancer cells induced a G2/M phase arrest, followed by apoptosis. A very recent study reported the development of an Aurora A PROTAC, JB170, showing that depletion of Aurora A results in strong S-phase arrest, which was not seen for the parent inhibitor TCS-7010, suggesting a non-catalytic scaffolding function of Aurora A during DNA replication [109]. JB170 induced a strong apoptotic phenotype in several cancer cell lines, which may open a new therapeutic window for the treatment of SWI/SNF-deficient cancers that depend on Aurora A [109].

The broad-spectrum tyrosine kinase inhibitor dasatinib was found to arrest the cell cycle during G1 and S phases through increased p21 and retinoblastoma protein expression, which resulted in a robust tumour reduction in ARID1A-mutated ovarian cancer [110]. This observation was confirmed in a second study, showing that small-molecule inhibition of the upstream regulators of p21, PI3K and AKT, also induced cell-cycle arrest and a lethal phenotype in ARID1A-deficient cell lines [49].

A quite intriguing synthetic lethal interaction was found for SMARCA4 and CDK4/6, suggesting that it may also be possible to exploit critically low levels of an oncogene for therapy. NSCLS and SCCOHT (small cell carcinoma of the ovary hypercalcemic type) cells are more sensitive to CDK4/6 inhibition with ribociclib or palbociclib than SMARCA4-proficient controls. This effect can be attributed—at least in part—to lower cyclin D expression levels in SMARCA4-deficient cancers, making them more susceptible to CDK4/6 inhibitors because their activity is already compromised [10, 111]. Interestingly, the low cyclin D levels in NSCLS seem to result from a combination of restricted chromatin accessibility of the *CCND1* locus and that of its transcriptional activator *JUN* [10, 111].

### Histone deacetylases

HDACs are frequently overexpressed in SMARCB1-mutant tumours and in ARID1A-deficienct cancers, making them sensitive to HDAC inhibitors. The pan-HDAC inhibitor panobinostat was able to induce MRT growth arrest, promote cellular senescence and inhibit self-renewal at low doses [112]. A second study showed that HDAC inhibition is more effective in combination with radiation when treating atypical teratoid/rhabdoid tumours [113]. ARID1A-deficient tumours depend on the activity of HDAC6, which erases the pro-apoptotic Lys120 acetylation mark in the DNA-binding domain of p53 [114]. Mechanistically, acetylation of Lys120 alters p53 DNA-binding specificity, favouring binding to response elements of pro-apoptotic target genes [115]. In addition, acetylation of several lysines in the C-terminal regulatory domain of p53 is crucial for p53 activation in general, by blocking ubiquitination sites and reducing non-specific DNA binding [46]. Accordingly, the selective HDAC6 inhibitor ricolinostat promoted an apoptotic response through p53 hyperactivation and thus improved the survival of mice bearing ARID1A-mutated ovarian tumours [114]. Another study on ARID1A-mutant cancer showed that HDAC2 and PRC2/EZH2 co-repressed the expression of apoptosis-promoting protein PIK3IP1 [116]. HDAC2 inhibition with SAHA re-localised HDAC2 to the cytoplasm and induced expression of PIK3IP1, which correlated with an improved prognosis for mice bearing ARID1A-mutated tumours [116].

### Poly ADP ribose polymerases

PARP enzymes involved in synthetic lethal interactions with SWI/SNF components are well-characterised drug targets and have been targeted with small molecules such as the highly potent PARP1 inhibitors talazoparib or veliparib, which block the NAD^+^ binding site [117]. In response to DNA damage, BRIT1 (BRCT-repeat inhibitor of hTERT expression) regulates the recruitment of SWI/SNF to DNA lesions [118]. This localisation is enhanced through phosphorylation of SMARCC2 by ATM and ATR kinases [118], and is supported by ARID1A, which interacts with double-stranded DNA breaks (DSBs) and regulates the DNA-damage checkpoint [119]. However, loss of ARID1A prevented ATR activation and DSB repair, generating a sensitivity to PARP inhibitors [119] as well as to ATR kinase inhibitors [98]. Talazoparib suppressed tumour growth of ARID1A-mutant cancers in a xenograft model by reducing expression levels of checkpoint kinase 1 and increasing levels of apoptosis marker caspase 3 [119]. In addition, ARID1A-mutant ovarian cancers are sensitive to a combined therapy of exogenous irradiation and PARP inhibition because of a reduced efficiency of the non-homologous end-joining (NHEJ) machinery to repair DNA lesions [120]. Lung cancer patients with ARID2 deficiency may also benefit from PARP inhibition, as PARP inhibitor veliparib has been shown to reduce cell survival of certain lung cancer cell lines [56].

### Ubiquitin-ligase/proteasome system and beyond

In MRT and renal medullary carcinoma cancer cells, an interesting link between SMARCB1 loss and the ubiquitin-ligase/proteasome system has been observed. MRT cells are sensitive to inhibition of the E3 ubiquitin ligase MDM2 (murine double minute 2) and its homologue MDM4/X. The MDM2 inhibitor idasanutlin, which blocks the MDM2-p53 interaction and hence proteasomal degradation of the latter, reduced tumour growth in an MRT xenograft model via upregulation of p53 [121]. These findings are consistent with the observation that MRT cells are highly sensitive to perturbations of the proteostasis programme and the p53/MYC-axis as shown by treatment with proteasome inhibitor ixazomib or the autophagy/lysosomal inhibitor chloroquine [122]. Moreover, it has been shown that SMARCB1-mutated renal medullary carcinoma are also dependent on the ubiquitin/proteasome system [123]. Proteasome inhibitors MLN2238 and bortezomib led to an accumulation of cyclin B1 as a result of an inactive E2 ubiquitin-conjugating enzyme, which triggered G2/M cell-cycle arrest [123].

Recent years have seen a surge of publications on targeting synthetic lethal interactions within the SWI/SNF interactome, not just involving the protein classes described in detail above. In addition, there are, for example, synthetic lethal interactions with the lysine demethylase of polycomb repressive complex 2 [124], downstream targets of c-MYC [125], components of the respiratory chain [126, 127] as well as with enzymes regulating the critical balance between glutathione and toxic reactive oxygen species [128] (see Table 2 and Fig. 5).

## Concluding remarks and outlook

The enormous impact of SWI/SNF chromatin remodelling complexes on chromatin biology, and thus on the regulation of almost all cellular pathways, has been gradually revealed over the last years. Ground-breaking discoveries ranged from how remodellers assemble their structures and organise chromatin accessibility, repair DNA defects or control gene expression, to distinct mechanisms that control cellular processes such as differentiation and proliferation. Perturbations of these fragile liaisons drive oncogenesis and promote tumour development. Mutations of SWI/SNF complexes occur in 20–25% of all human cancers, making them prime targets for therapeutic intervention [13]. Because every tumour differs in its genetic or epigenetic profile, there is a great need for new medicines and personalised therapies to successfully treat cancer patients. Choosing the right drug is thus highly context-dependent, and therapeutic concepts must be assessed on an individual basis.

At first glance, SWI/SNF inhibition seems counterintuitive, given its tumour suppressor functions. Indeed, inactivation of certain SWI/SNF subunits may actually promote cancer development. A very recent paper provided intriguing insights into how SWI/SNF cancers may be successfully targeted [129]. This study on the nematode *Caenorhabditis elegans* suggested that it is all about the right balance—as with so many things in life. Cancer cells depend on an incomplete inactivation of SWI/SNF genes to maintain their full oncogenic potential. A partial loss-of-function eliminates the intrinsic tumour suppressor properties of SWI/SNF complexes, leading to aberrant proliferation and differentiation, whereas the complete loss of SWI/SNF activity results in cell division arrest during cell development in the *C. elegans* model [129]. The ATPase subunit is a good example to illustrate this phenomenon. High functional levels of the ATPase subunit are required to maintain its tumour suppressor activity. Mutation of this gene can result in lower functional levels, but, importantly, the residual SWI/SNF components are able to sustain cell proliferation [129]. Similarly, in malignant rhabdoid tumours, SMARCB1 loss reduces the ability to bind to typical enhancers, but not to super-enhancer sites, indicating that the residual SWI/SNF subunits are sufficient for tumour progression and survival through formation of ncBAF complexes [21, 23].

In recent years, immense progress has been made in the identification and validation of targets to treat SWI/SNF-mutant cancers (Fig. 5) as well as in the development of selective and potent bromodomain inhibitors for directly targeting SWI/SNF function (Fig. 4) [80, 89, 90, 93]. It came somewhat as a surprise, though, that these bromodomain inhibitors failed to display significant antiproliferative effects. Again, it seems that the non-targeted SWI/SNF domains are sufficient to ensure sustained cell survival. But, with the advent of the PROTACs technology, which enabled the development of these inhibitors into specific degraders of entire SWI/SNF subunits, several proof-of-concept studies have now shown that it is indeed possible to induce synthetic lethality with chemical probes targeting bromodomains [100, 101]. The beauty of this technology is that it offers new targeting opportunities that are still in their infancy. The most intriguing aspect is undoubtedly that it is now, in principle, possible to target ‘undruggable’ SWI/SNF components, for example, specific scaffolding modules, by targeting associated domains for which inhibitors exist. Many SWI/SNF complex subunits contain interaction modules of yet unknown function. Recent structural studies have, for example, identified a binding groove in the BRK domain of SMARCA4 that may be druggable [130], and more potential targets may emerge in the future.

Targeting the SWI/SNF regulatory network may also be a therapeutic option to induce synthetic lethality, but this requires a more detailed understanding of the intricate mechanistic details of this network, which is currently still lacking. Several studies showed (de)phosphorylation mechanisms in the DNA-damage response, indicating that multiple protein kinases are involved in SWI/SNF regulation [131, 132]. There is a plethora of available kinase inhibitors that could be repurposed for investigating the phosphorylation state of SWI/SNF and its functional consequences. Again, the development of PROTACs, as in the case of the recently developed Aurora A degrader [109], seems to be the way forward to achieve improved therapeutic effects. Another potential future strategy may involve targeting protein–protein interactions with allosteric modulators in order to induce conformational changes within specific SWI/SNF subunits and/or alter the overall assembly of the complex and thereby change the biological binding profile.

The combined efforts of cell biologists, medicinal chemists and structural biologists will hopefully see some of those strategies come to fruition in the near future and provide tailor-made compounds for targeting SWI/SNF complexes in different types of cancer.
